# Strength of Crowd (SOC)—Defeating a Reactive Jammer in IoT with Decoy Messages

**DOI:** 10.3390/s18103492

**Published:** 2018-10-16

**Authors:** Savio Sciancalepore, Gabriele Oligeri, Roberto Di Pietro

**Affiliations:** Division of Information and Computing Technology, College of Science and Engineering, Hamad Bin Khalifa University, Doha 5825, Qatar; goligeri@hbku.edu.qa (G.O.); rdipietro@hbku.edu.qa (R.D.P.)

**Keywords:** IoT, distributed systems, anti-jamming protocols, reactive jamming, proactive jamming, experimentation

## Abstract

We propose Strength of Crowd (SoC), a distributed Internet of Things (IoT) protocol that guarantees message broadcast from an initiator to all network nodes in the presence of either a reactive or a proactive jammer, that targets a variable portion of the radio spectrum. SoC exploits a simple, yet innovative and effective idea: nodes not (currently) involved in the broadcast process transmit decoy messages that cannot be distinguished (by the jammer) from the real ones. Therefore, the jammer has to implement a best-effort strategy to jam all the concurrent communications up to its frequency/energy budget. SoC exploits the inherent parallelism that stems from the massive deployments of IoT nodes to guarantee a high number of concurrent communications, exhausting the jammer capabilities and hence leaving a subset of the communications not jammed. It is worth noting that SoC could be adopted in several wireless scenarios; however, we focus on its application to the Wireless Sensor Networks (WSN) domain, including IoT, Machine-to-Machine (M2M), Device-to-Device (D2D), to name a few. In this framework, we provide several contributions: firstly, we show the details of the SoC protocol, as well as its integration with the IEEE 802.15.4-2015 MAC protocol; secondly, we study the broadcast delay to deliver the message to all the nodes in the network; and finally, we run an extensive simulation and experimental campaign to test our solution. We consider the state-of-the-art OpenMote-B experimental platform, adopting the OpenWSN open-source protocol stack. Experimental results confirm the quality and viability of our solution.

## 1. Introduction

Wireless Sensor Network (WSN) are today considered the enabling communication framework for several scenarios, including Internet of Things (IoT), Machine-to-Machine (M2M), Device-to-Device (D2D) and Supervisory Control And Data Acquisition (SCADA) systems [1]. Unfortunately, the openness of the radio spectrum makes these scenarios prone to several cybersecurity attacks, with Denial of Service (DoS) being probably the most disruptive one. In this context, one of the most effective DoS techniques is jamming, consisting of malicious transmissions realized with the aim of only disrupting legitimate communications [2,3].

Wireless jamming can be achieved through a large variety of strategies, according to the communication pattern under attack. An early classification takes into account their behavior against the signal to be disrupted. Jammers disrupting the communications on one or multiple random adjacent frequencies are called proactive. Indeed, the jammer decides in advance the frequencies to be jammed in a given temporal slot, and it transmits random noise independently of the presence of a signal. However, the simplicity in the detection of these kinds of jammers inspired the rise of new, smart techniques, more difficult to be identified and defeated. This is the case for the reactive jammers, able to continuously listen on the wireless channel seeking for the presence of upcoming communications and capable of quickly switching to transmit an intentional interference as soon as the presence of a new radio packet is detected. Being active and effective only for a small portion of time, such attacks are not only very hard to detect, but also extremely challenging to mitigate.

Even if some contributions in the literature provide effective mechanisms to communicate even in the presence of reactive jamming (see Section 2 for a comprehensive overview), these solutions do not consider powerful adversaries, able to disrupt the operation of an IoT network without any spatial limitation. Where such a requirement is considered, the cited solutions require the migration to innovative and customized transmission and modulation techniques, or they impose the modification of the standard information encapsulation and decapsulation processes. Indeed, they cannot be implemented in application scenarios in which neither the hardware processing chain of the devices, nor the underlying technologies at the lowest layers of the protocol stack can be modified.

**Contribution:** We propose Strength of Crowd (SoC), a distributed protocol suitable for IoT constrained devices, that guarantees the delivery of a message to all the nodes in a wireless network even in the presence of a wide-band, spatially unlimited, global eavesdropping reactive and proactive jammer, disrupting up to *A* out of *F* available frequencies. SoC leverages the inherent parallelism arising from massive nodes’ deployment (typical of some IoT scenarios) to generate decoy transmissions and hence confusing the adversary about the real ones. Specifically, SoC is rooted on the transmission of decoy packets by legitimate devices in the network, making just probabilistic the outcome of the jamming of a data message.

The performance of SoC has been studied and validated through both extensive simulations and a real implementation, assuming the parameters of a typical IoT scenario. In particular, we demonstrate the feasibility and practicality of SoC by implementing it in real IoT devices such as the OpenMote-B recently released on the market. Our experimental results demonstrate that, assuming eight nodes in the network, five available frequencies and a jammer spanning 80% of the available spectrum, SoC is able to guarantee the delivery of a message to the entire network in about 300 slots (3 s), while traditional channel hopping mechanisms would fail to deliver the message under such adversary assumptions. Finally, we highlight that, contrary to the majority of the solutions available in the literature, SoC is a standard-compliant distributed algorithm, easy to integrate within any protocol stack, not requiring any modification, neither to the underlying IEEE 802.15.4 technology, nor to the transmission/reception chain at the physical layer.

**Paper organization:** The paper is organized as follows: Section 2 reviews related work; Section 3 introduces the system and the adversary model assumed in this work, while Section 4 details the proposed SoC scheme. Section 5 provides both simulations and experimental assessment of the proposed approach, while Section 7 presents a comparison with the state of the art and some remarks. Finally, Section 8 tightens conclusions and illustrates further research directions.

## 2. Related Work

Mitigating the presence of a jammer in WSNs is a well-known topic. While some recent contributions focused on detecting a reactive jammer [4,5,6], only a few of them proposed solutions to still allow communications in such a scenario.

The authors in [7] introduced LAPSE, a link quality-aware path selection algorithm that maximizes the link quality when choosing alternative paths in the presence of jammers disrupting a fraction of the network.

A Decision Fusion (DF) algorithm based on a Rician link model and partially unaware link jamming has been proposed in [8]. DF algorithms send local decisions to a DF center in order to take global actions. The authors proved that the proposed rules are effective to mitigate the presence of the jammer. It is worth noting that the algorithm is centralized; thus, forwarding decisions to the DF center under a wideband jammer could be hard to achieve.

A distributed and dynamic solution to selective jamming in TDMA-based WSNs was presented by [9]. The authors proposed JAMMY, a solution that changes the slot utilization pattern at every superframe, thus making it unpredictable to the adversary. The proposed solution is fully decentralized, as sensor nodes determine the next slot utilization pattern in a distributed and autonomous way. Results from performance analysis of the proposed solution show that JAMMY introduces negligible overhead, yet allowing multiple nodes to join the network in a limited number of superframes.

The authors in [10,11] provided solutions to still allow communications in the presence of a reactive jammer that does not detect the presence of some transmission because of the distance from its source. Thus, their model assumed an adversary that is spatially limited and that tackles only the communication on a given link.

In [12], the authors proposed an intelligent solution to collaboratively map the WSN jammed region and avoiding traffic through such an area. The proposed technique finds the nodes inside the jammed area without extensive flooding, thus reducing the traffic inside the jammed region substantially (contrary to [13]), while retaining its basic operation around the boundary. The proposed solution is reliable and with a reduced traffic overhead of about 20–25%. However, the adversary model assumes that the jammer is spatially limited, while the adversary assumed in our work is supposed to be aware of the channels used throughout the whole network.

A survey about attacks and defense strategies is proposed by [14]. The authors reported different jamming attacks that may be employed against a WSN. To cope with the problem of jamming, they discussed a two-phase strategy involving the diagnosis of the attack, followed by a suitable defense strategy. One approach is to simply retreat from the jammer, which may be accomplished by either spectral or spatial evasion. The second approach aims to compete more actively with the jammer by adjusting resources, such as power levels and communication coding, to achieve the communication.

The authors in [15,16] specifically focused on mitigating reactive jamming. They proposed a new scheme to deactivate jammers by efficiently identifying all trigger nodes, whose transmissions invoke the jamming nodes. Such a trigger-identification procedure can work as an application-layer service and benefits many existing reactive-jamming defending schemes. However, the trigger identification scheme is based on decisions made only by the base stations, thus configured as a centralized solution. In addition, the adversary model assumes that the jammers focus only on a limited area of the network.

When the jammer has the ability to interpret data link layer protocols, it becomes as energy-efficient as legitimate nodes. The authors in [17] presented a comprehensive survey on different sophisticated jamming attacks based on the MAC layer. Techniques used to defeat each one of the intelligent jammers are classified based on the knowledge capacity of MAC protocol rules.

A general overview of critical issues about jamming in WSNs was presented by [3]. The authors provided an overview of the communication protocols adopted by WSN deployments, and they highlighted the characteristics of contemporary WSNs that make them susceptible to jamming attacks, along with the various types of jamming that can be exercised against WSNs.

The authors in [18] provided a mechanism to divide a WSN under attack by a jammer into different zones as per the severity of jamming experienced by various nodes of the network. Previous approaches were able to map the geographical extent into only two zones: “jammed” and “not jammed”, but at the same time, they were vulnerable to information warfare attacks, as they all were required to communicate, even under a jamming attack. Instead, this solution is based on a centralized approach, where the mapping is done by the base station through hull tracing of jammed nodes as per their pre-calculated jamming indices.

Jamming of WSNs base stations was considered in [19]. To tackle base-station jamming, replication of base stations, as well as jamming evasion, by relocation to unjammed locations, have been proposed. The authors introduced Honeybees, an energy-aware defense framework against base-station jamming attack in WSNs. Honeybees efficiently combines replication and evasion to allow WSNs to continue delivering data for a long time during a jamming attack considering three different jamming strategies: reactive, proactive and hybrid.

The authors in [20] proposed an anti-jamming communication system that allows communication in the presence of a broadband and high power reactive jammer. The proposed system transmits messages by harnessing the reaction time of a reactive jammer, but it requires the modification of the MAC protocol in order to include the information right after the end of the physical layer headers. This makes the scheme not compliant with any IoT standard.

Finally, it is worth noting that in this work, we consider a scenario in which the information needs to be broadcast from one originating node to all the nodes in the network. Recently, approaches such as [21] were pushing toward low-cost adaptive techniques for data dissemination, able to reduce the amount of bandwidth required for data dissemination consistently. To cite an example, the Adaptive Monitoring Dissemination (ADMin) open-source framework proposed in [21] efficiently adapts, in place, the rate at which IoT devices disseminate monitoring streams to receiving entities based on the evolution and variability of the metric stream. Indeed, these approaches, when coupled with SoC, could allow one to spread the information by requiring fewer time-slots. However, not assuming any mechanism to help the spreading of the information, the provided results still represent an upper bound on the overall broadcast delay.

To sum up, as will be explicitly discussed in Section 7, none of the solutions discussed above provide a standard compatible approach, requiring no modifications at the physical and the MAC layer protocols and being able to overcome a geographically unlimited wide-band reactive jammer.

## 3. System and Adversary Models

In this section, we introduce both the system and the adversary model assumed throughout our paper, as well as some basic preliminary assumptions and related motivations.

### 3.1. System Model

**Figure 1:**
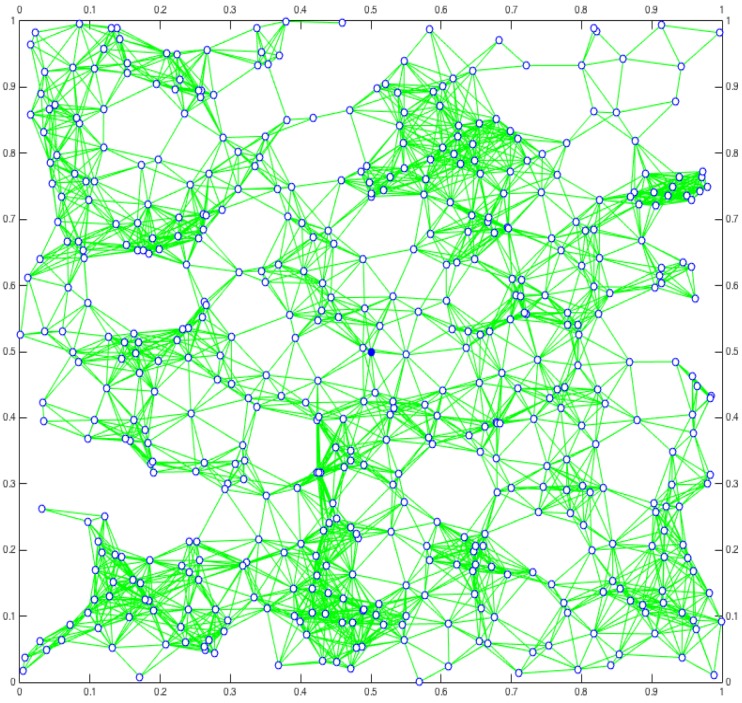
A typical network deployment where each node experiences an average number of neighbors of 10 and the network is fully connected. The central blue node—coordinates (0.5,0.5)—is the initiator of the message broadcasting.

We consider a wireless network constituted by N=512 nodes uniformly distributed in a squared area of unitary side. Each node features a wireless radio, and it is able to communicate in a spectrum of F=32 frequencies. All the nodes behave in the same way, and their transmission range is such that it guarantees the full network connectivity. Indeed, the minimum number of neighbors *n* in order to guarantee the full network connectivity is Θ(ln(N)), i.e., n>6 [22]. Therefore, when the number of neighbors is greater than n>10, we can practically assume that the network is fully connected [23,24]. Figure 1 shows a typical network deployment considered in our scenario.

We assume that the nodes are loosely time synchronized and the communications take place on a time-slot basis [25]. At each time-slot, the node $n_{i}$, with i∈{1,…,N}, can transmit or receive by tuning its radio on a random frequency $f_{j}$, with j∈{1,…,F}. We assume the slot duration *T* to be large enough to transmit a packet and receive the corresponding acknowledgment from the receiver, consistently with the majority of MAC standards for wireless networks [25].

We also assume that each node in the network is able to produce new information, e.g., by sensing the surrounding environment through its sensors. Then, the node wants to spread (and replicate) the information to all the nodes in the network.

### 3.2. Adversary Model

In this work, we consider a very powerful adversary, namely E, featuring the following characteristics.

**Reactive behavior:**E waits for a signal to be transmitted on the eavesdropped spectrum. As soon as a new message is detected (i.e., through the identification of the physical-layer preamble), it starts jamming the packet by injecting random Gaussian noise.

**Proactive behavior:**E chooses *A* out *F* frequencies, and it transmits random Gaussian noise over them at the same time.

**Spatially unbounded:**E is spatially unlimited, i.e., it can listen to all the transmissions in the network, independently of the distance between its physical location and the location of the transmitter. Note that this is a strong assumption from the attacker perspective, given that in reality, E would be able to listen only to transmissions that happen in its coverage area. However, this conservative stance allows us to obtain results that, from the perspective of the legitimate nodes, represent a lower bound on the achieved performance.

**Global eavesdropper:**E is able to detect and listen to any communication in the network, independently of the frequency $f_{j}$ used for the communication.

**Multi-frequency jammer:**E is able to actively operate simultaneously on a subset *A* out of *F* available frequencies that can be used by the IoT devices to communicate.

## 4. The SoC Protocol

In the section, we introduce SoC, the distributed protocol able to guarantee the message delivery to all the nodes in the network in the presence of both proactive and reactive jammers. Section 4.1 introduces the rationale of the scheme, while the details of SoC are discussed in Section 4.2.

### 4.1. SoC Rationale

Jamming mitigation throughout broadcasting the message to all the nodes in the neighborhood has already been proposed by some authors [26,27,28]. Nevertheless, our approach is different. Indeed, while other contributions assume a proactive jammer, i.e., a jammer transmitting on random frequencies, in this work, we also consider a reactive jammer with full network visibility. Given the characteristics of the jammer, we propose a protocol that exploits the reactivity of the jammer to saturate its jamming capabilities. Since the jammer is able to listen to all of the *F* available frequencies, but to jam up to *A* out of the total *F* available frequencies, we suggest exploiting all the nodes of the network to transmit decoy messages with the purpose of hiding the real transmissions to the jammer. Indeed, given its tight time constraints, the jammer has to make a decision to jam or not the packet very quickly and by looking only at the packet’s preamble [20]. Therefore, the jammer is not able to discriminate in advance between a real and a decoy packet, and thus, under a conservative stance, it has to jam both of them.

It is worth noting that a simple frequency hopping would be ineffective in the adversary model assumed in this work. In fact, given that the jammer is able to listen to all frequencies available for the communication, it could easily intercept the transmission of the information and immediately disrupt the packet. To increase the chances of successful delivery under a simple frequency hopping, each legitimate node would be forced to increase its sampling frequency (e.g., by acquiring more often new data from the surrounding environment), thus drastically increasing its energy consumption.

### 4.2. Details of SoC

The pseudo-code of the SoC protocol is reported in Algorithm 1.

**Algorithm 1:** Pseudo-code of SoC

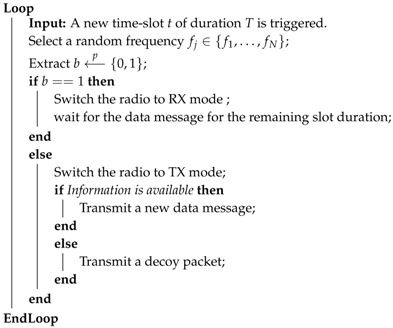



The SoC protocol assumes that nodes are loosely time synchronized on a time-slot basis. This might be achieved by adopting a network-wide synchronization protocol [29].

At the beginning of each time-slot, each node decides the operating frequency $f_{j}$ among the *F* available in the radio spectrum. Next, the node assigns either one or zero to *b* with probability *p*, where *p* is a random variable uniformly distributed. Without loss of generality, we consider the node as a receiver when b==1, while we consider the node as a transmitter when b==0. Thus, when b==1, the node switches the radio to reception mode (RX mode) and waits for a packet to be received on the selected frequency $f_{j}$. Conversely, when b==1, the node switches the radio to the transmit mode (TX mode) and it transmits either the data message, if new information is available in its buffer, or a decoy packet, if the information message has not been received yet.

It is worth noting that the probability *p* to be either a receiver or a transmitter affects both the speed at which the message propagates through the network and the resiliency of SoC against the jammer. In fact, if the node is a receiver, it increases the probability that the information is propagated. If the node is a transmitter, it increases the chances for the neighbors to deliver the message, by confusing the adversary, adding one more (decoy) transmission to the radio spectrum.

**Figure 2:**
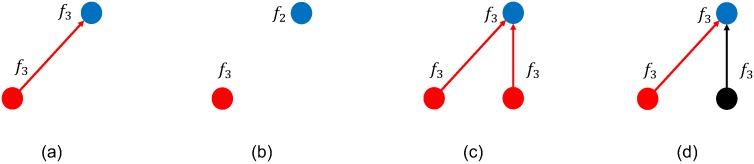
Communication scenarios. Red circles represent nodes with the message; blue circles represent receiving nodes; and finally, black circles represent transmitters of decoy messages. At each round, the message delivery might be successful (**a**) or fail. When the message is not delivered, it might be due to the transmitter and receiver being on different frequencies (**b**), a collision due to two or more message transmitters (**c**) and finally, a collision due to a decoy transmitter (**d**).

Moreover, message propagation depends on other factors as depicted in Figure 2. Indeed, the transmitter and the receiver could select different frequencies for the respective radio operations (Figure 2b), or a transmitted data message might collide with another one (Figure 2c) or with a decoy (Figure 2d). A transmitted message propagates in the network when no collisions happen and both the transmitter and the receiver are using the same frequency (Figure 2a); otherwise, the message propagation fails (Figure 2b–d).

## 5. Performance Assessment

In this section, we analyze the performance of the proposed SoC protocol resorting to both simulations and real experimentation. Both of the scenarios assume first a benign scenario, with the aim of establishing a benchmark, i.e., the performance of the proposed scheme in ideal conditions. Then, an adversary with increasing jamming capabilities is introduced to show the performance in a real scenarios. Simulations have been performed with MATLAB© R2018a and a DELL precision 5720 workstation, equipped with two i7 processors working at 3.60 GHz, 32 GB of RAM and 2 TB of HDD memory.

### 5.1. Benign Scenario

We start our performance analysis from a benign scenario where the adversary is not present, with the aim of establishing a performance benchmark for the proposed approach. Our benign scenario is constituted by N=512 nodes uniformly distributed over an area of dimensions [1×1] units. Each node has a transmission range of 0.09 units, guaranteeing an average of 10 neighbors, hence achieving the full network connectivity. We also considered a spectrum of available frequencies F=32. Finally, we consider only one initiator node, i.e., a node with a message to be delivered to all the network, placed at the center of the network, i.e., [0.5,0.5].

**Definition** **1.** *We define broadcast delay $B_{d}$ as the number of time-slots requested to deliver the message to the 95% of the nodes in the network.*

**Figure 3:**
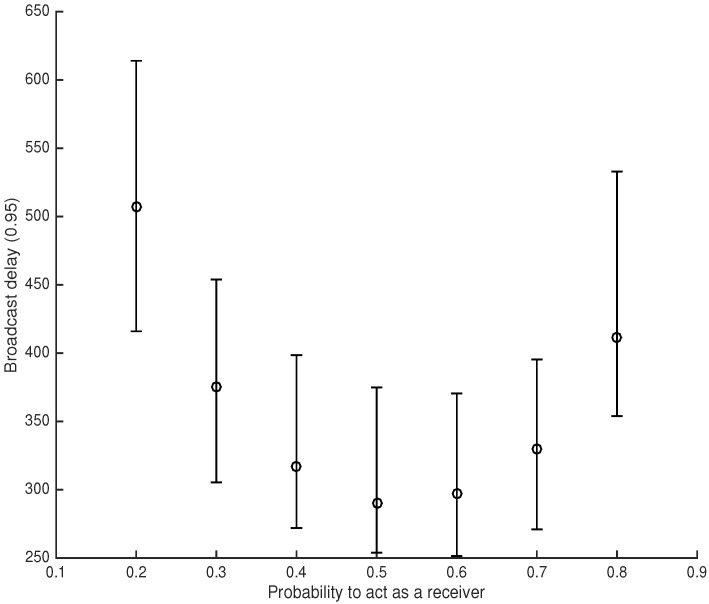
Error bars show quantiles 5, 50 and 95 associated with the broadcast delay to deliver the message to 95% of the nodes in the network N=512.

The error bars in Figure 3 show the quantiles of 5, 50, and 95 associated with the broadcast delay as a function of the probability *p* to act as a receiver (recall Algorithm 1). We observe that the broadcast process is affected by large delays when either p<0.3 or p>0.7. The protocol guarantees the best performance (i.e., the minimum broadcast delay) when p≈0.5. This is indeed the best trade-off to guarantee each node to act either as a receiver or as a transmitter. When the protocol is biased towards either transmission (p<0.5) or reception (p>0.5), the message propagation is significantly delayed due to the presence of too many transmitters and receivers.

### 5.2. Scenario with Reactive Jammer

**Figure 4:**
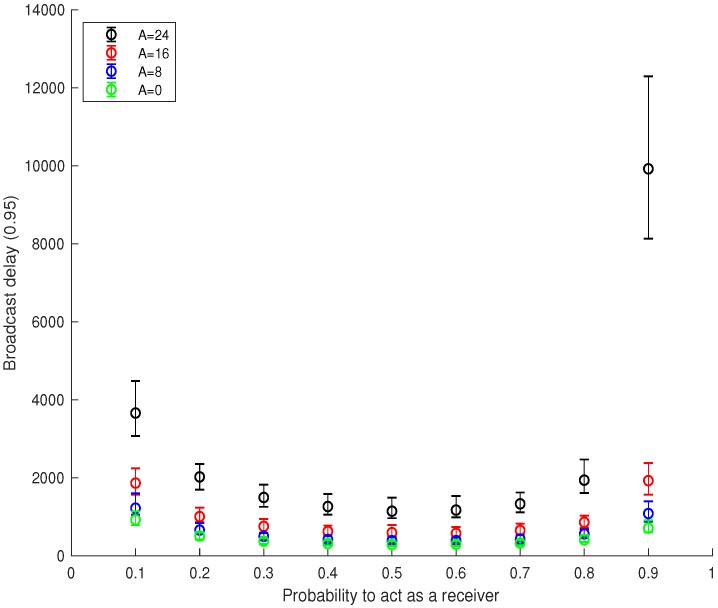
Performance of Strength of Crowd (SoC) in the presence of a jammer assuming different jammed frequencies (*A* out of *F*) and the probability to act as a receiver spanning between 0.1 and 0.9.

We consider the same network configuration of Section 5.1, i.e., N=512 nodes uniformly distributed over an area of [1×1] units. Our jammer is a global eavesdropper able to jam up to *A* out of *F* communications in the radio spectrum. The error bars in Figure 4 show the quantiles of 5, 50 and 95 associated with the broadcast delay assuming the number of jammed frequencies spanning between zero and 24 out of the 32 available frequencies. The trends of all the configurations are consistent with those already presented in Figure 3. Indeed, the jammer introduces a constant delay in message propagation depending on the fraction of the jammed radio spectrum. We observe only one particular case where the jammer significantly delays the broadcast process, i.e., when A=24 and p=0.9. That specific case can be explained by observing that the network is mostly constituted by receivers, and the jammer can easily prevent the message propagation due to the absence of transmitters of either the real data message or decoy packets. The latter case actually confirms the importance of decoy packets to deceive a reactive jammer. Moreover, we observe that the optimal value for the *p* parameter is still 0.5, guaranteeing a perfect trade-off in the number of transmitters and receivers. Finally, it is worth noting that the SoC protocol is able to deliver the message to 95% of the network even in the presence of a jammer able to disrupt 75% of the communications (A=24 frequencies out of 32) with a broadcast delay that is four-times the one incurred under benign conditions (A=0).

In order to highlight the efficiency of SoC in the presence of jamming, we report the broadcast delay variations in Table 1. For each scenario, we consider the ratio of the broadcast delay with respect to the benign case (A=0). It is worth noting that the broadcast delay is not significantly affected by the jammer; indeed, even considering the most powerful adversarial configuration (reactive adversary able to jam 75% of the communications), SoC is still able to broadcast a message to all the nodes in the network in approximately four-times the broadcast delay of a benign scenario, meaning a period of time of about 14.7 s, where we assume a slot time duration of 10 ms, consistent with the IEEE 802.15.4-2015 standard, adopted by most of the IoT technologies (e.g., Bluetooth and ZigBee) [25].

### 5.3. Experimental Assessment in the Presence of a Proactive Jammer

To provide further insights and to demonstrate the effective feasibility of the proposed solution, we implemented SoC in a real IoT platform. Specifically, we considered the OpenMote-B experimental platform, i.e., the state-of-the-art hardware board for real experimentation and rapid prototyping of IoT algorithms and solutions [30,31]. The board features a 32-MHz CC2538 SoC, 512 kB of ROM and 32 kB of RAM, as well as the integration with four sensors, i.e., temperature, humidity, light and acceleration. As for the operating system, we selected the well-known OpenWSN, consistent with other related work on IoT and Industrial Internet of Things (IIoT) [32,33,34], since it integrates a slotted channel access mechanism and the widely-accepted IEEE 802.15.4 standard operating in the TSCH mode [35].

**Figure 5:**
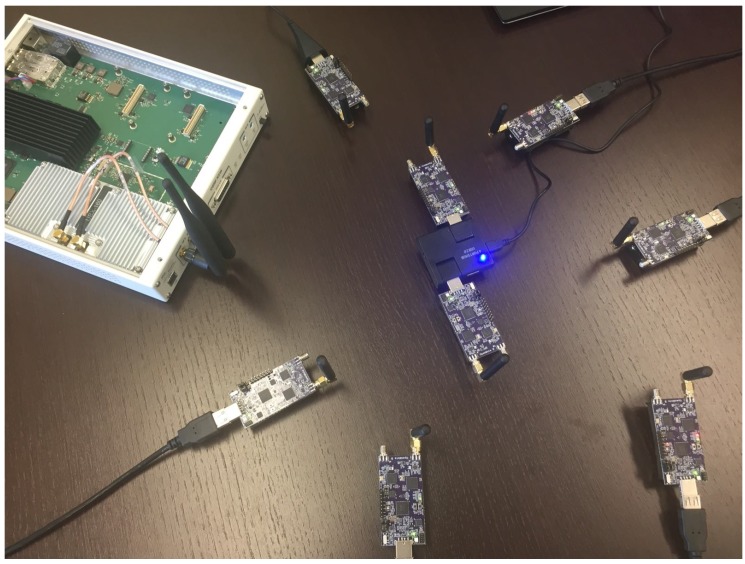
Our deployment of nodes and the jammer.

As for the jamming devices, we used the state-of-the-art USRP X310 Software-Defined Radio (SDR), integrated with the powerful UBX160 daughterboard (https://www.ettus.com/product/details/X310-KIT), consistently with other related work dealing with jamming [20,36,37]. The UBX160 has an operating bandwidth able to span from 10 MHz–6 GHz and a maximum signal bandwidth of 160 MHz. Finally, we adopted GNURadio (https://www.gnuradio.org/) as the software for configuring the SDR and managing the jammer. Figure 5 shows our deployment with the nodes and the jammer.

First, we report in Table 2 the ROM and RAM footprint of our implementation in the OpenWSN protocol stack.

Note that the implementation of SoC is very lightweight, both in terms of ROM and RAM footprint, requiring less than 1 kB of code and a negligible amount of RAM, dedicated only to the storing of some variables for its state. Therefore, it is particularly suitable for very constrained devices, having a small amount of available RAM.

Then, we configured a fully-connected network with a total number of N=8 IoT devices and with a total number of F=5 frequencies for the communication. Conversely, the proactive jammer has been implemented by using multiple SDRs (one per frequency), by increasing the portion of the jammed spectrum, from 20% up to 80%, in line with other related works available in the literature. The proactive jammer injects random noise on a given frequency set, independently of the presence of any communication in the channel.

**Figure 6:**
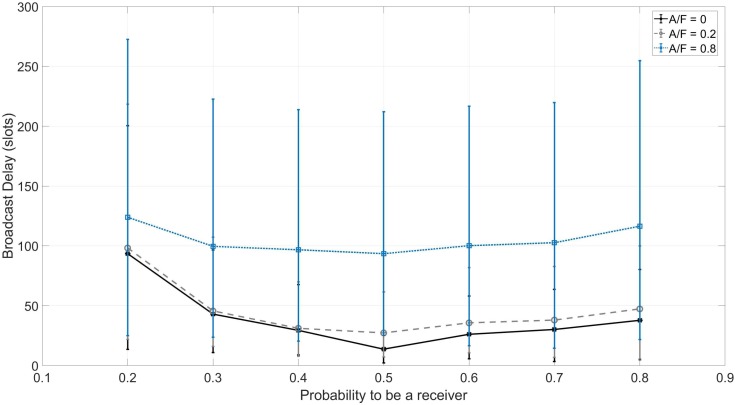
Experimental results with *F* = 5 and *N* = 8.

Each configuration has been repeated 40 times. The results are reported in Figure 6, along with the 95% confidence interval.

As expected, the broadcast delay increases as the capabilities of the jammer increase. Table 3 shows the average broadcast delay variations with respect to the benign scenario. We highlight how SoC is able to broadcast a message in the presence of a proactive jammer disrupting 80% of the radio spectrum (four frequencies out of five) experiencing a broadcast delay that is only seven-times longer than the one of the benign scenario.

We remark that the aim of this experimental campaign is two-fold: (i) to demonstrate the feasibility of SoC when run on real, constrained, IoT devices, and (ii) to prove that SoC guarantees the message broadcast in a real scenario, even when dealing with a powerful jammer. Finally, we recall that simulated results are significantly better, i.e., broadcast delay variation with respect to the benign scenario is 4.09 (Table 1): this is mainly due to the fact that simulations involve more nodes, and therefore more entities, participating in the broadcast process.

## 6. Energy Consumption

**Figure 7:**
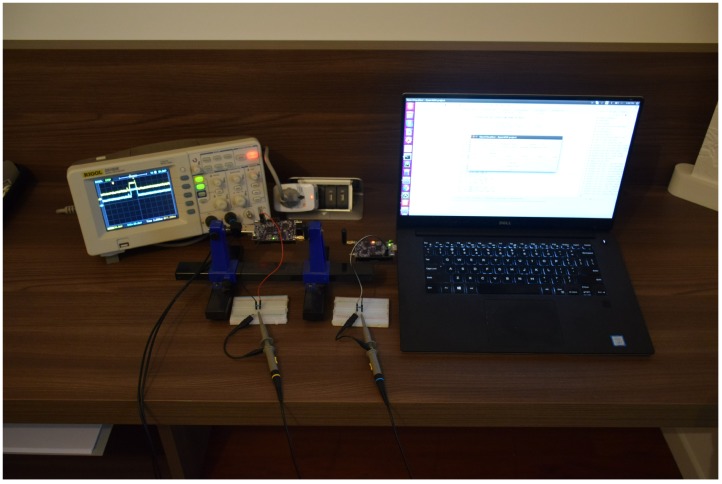
Measurement scenario: We used a two-channel oscilloscope to measure the voltage drop at both the transmitter and receiver of a 1 Ω probe resistor placed in series with the CC2538 chipset.

To measure the current drawn by the SoC protocol, we used a RIGOL DS1052E digital oscilloscope, by sampling the voltage drop to the terminals of a 1 Ω probe resistor bridging the pins in series with the CC2538 chipset. The RIGOL DS1052E has a vertical resolution of eight bits, and the vertical range has been set to 20 mV/div, while the horizontal range has been set to 2 ms/div. We sampled several runs of the protocol and subsequently exported the data to MATLAB for the analysis. The measurement scenario is depicted in Figure 7.

**Figure 8:**
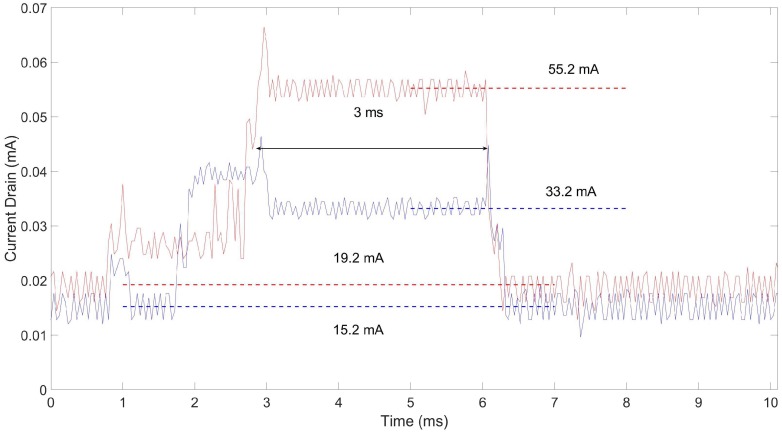
Current drained by the OpenMote-B device: blue line represents the receiving device while the red line represents the transmitting device.

Figure 8 shows the voltage drop associated with the TX (red line) and RX (blue line) activities during the execution of the SoC protocol, within the duration of one slot. In order to evaluate the overall energy consumption, we consider the different contributions as depicted in Table 4 from the data sheet [38].

We start our analysis from the device steady-states, i.e., t≤2 ms and t≥6 ms, in Figure 8. We observe the average values of 15.2 mA and 19.2 mA for the blue (RX) and red (TX) curves, respectively. Indeed, the energy consumption at the receiver can be summarized as: 13 mA (CPU) + 2 mA (2 LEDs) ≈ 15 mA. On the other hand, we have 13 mA (CPU) + 3 mA (3 LEDs) + 3 mA (USB UART) ≈ 19 mA at the transmitting side. We recall from Figure 7 that the transmitter is connected to the laptop. Then, we have a transient period for both the transmitter (26 mA for 2 ms) and the receiver (40 mA for 1 ms) due to the radio core preparing for the transmission/reception of a new message. Finally, we have the actual transmission/reception of the message lasting for 3 ms. Packet reception requires 20 mA, summing up to an overall consumption of 15 mA + 20 mA ≈ 35 mA; conversely, packet transmission involves one more LED (1 mA) and 34 mA due to the radio transmission process with a transmitting power of 7 dBm summing up to 19 mA + 1 mA + 34 mA ≈ 54 mA.

We observe that the theoretical values slightly differ from the measured ones for less than 1 mA. Such an error might be due to several measurement factors such as the probe resistor value, oscilloscope readings, temperature, etc.

The energy consumption in the slot, namely *E*, can be computed by integrating the instantaneous current drain i(t) over the time duration *T* of the slot and multiplying it by 3.3 V, i.e., the voltage of the OpenMote-B board [38], yielding:

(1) $$E\left(\mathrm{mJ}\right)=3.3V\int_{0}^{T}i\left(t\right)dt$$

In order to evaluate the actual RX/TX process consumption, we removed from the previous analysis all the consumption factors related to debug components, such as the LEDs and the USB UART, i.e., 2 mA at the receiver side and 6 mA at the transmitting side (with 1 mA more during the actual transmitting process). Therefore, the RX procedure consumes about 38 mA × 1 ms = 38 mJ for the radio core preparation and 18 mA × 3 ms = 54 mJ for the reception process, summing up to 92 mJ. Conversely, the transmission procedure consumes 20 mA × 2 ms = 40 mJ for the radio core preparation and 27 mA × 3 ms = 81 mJ for the transmission procedure, summing up to 121 mJ. Considering also the consumption in the remaining part of the slot (lasting for 10 ms), where only the CPU is active, we have that the TX slot consumes 186 mJ, while the RX slot consumes 170 mJ.

To obtain the energy consumed by a node during the whole protocol, we need to consider the overall number of slots necessary to reach the full coverage of the network. As depicted in Figure 6, the protocol takes a number of slots equal to $N_{all}$ to complete the spreading of the information message throughout the whole network, depending on the portion of the spectrum disrupted by the adversary. During this time, each node spends a percentage of the slots equal to $N_{all}/100\times p_{RX}$ in receiving mode, while the remaining slots will be spent in transmission mode, transmitting decoy or information messages. Thus, the overall mean energy consumed by this node can be computed as follows:

(2) $$E_{TOT}\left[\mathrm{mJ}\right]=\left(N_{all}/100\times p_{RX}\right)\times E_{RX}+\left(N_{all}/100\times p_{TX}\right)\times E_{TX},$$

where $E_{RX}$ and $E_{TX}$ refer to the energy consumed in a single RX and TX slot, respectively.

**Figure 9:**
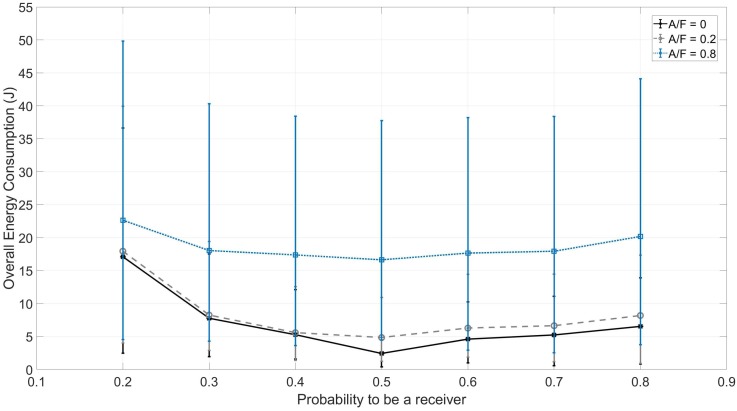
Experimental energy consumption with *F* = 5 and *N* = 8.

Results are shown in Figure 9, with reference to the experimental results discussed in Section 5.3.

As the energy consumption is directly related to the duration of the protocol, the scenario where the adversary jams most of the channels is the most energy consuming. In this context, the configurations where the probability to be a receiver is lower are the most energy consuming, due to two main reasons. First, the time necessary to achieve full network coverage is the highest. Second, a node spends most of the time transmitting messages on the wireless radio interface, and the energy consumption in a transmission slot is slightly higher than the one in a reception slot (this is related only to the operation of the OpenMote-B hardware board, while this is not always true for other hardware boards). At the same time, the configuration characterized by a probability to be a receiver having a value $p_{RX}=0.5$ is not only less time-consuming, but also less energy-consuming.

Given that a typical manganese/alkaline AA cell is rated at about 2.4 ampere-hours, if we assume 1.5 volts on average, we have approximately 3.84 watt-hours, equivalent to 13,824 Joules of storage capacity [39].

Thus, even considering the most energy-consuming scenario in which the adversary jams 80% of the spectrum and the node is configured with a probability to be a receiver of 0.2%, SoC drains approximately 0.16% of the whole battery capacity. By choosing the probability value as $p_{RX}=0.5$, it is possible to further reduce the consumption down to 0.12% of the battery capacity.

Finally, it is worth noting that, considering the same time duration *T* of the protocol and the OpenMote-B hardware platform, transmitting decoy messages is more energy consuming with respect to simply listening for the message. In fact, in case the node still has not received the information message, without SoC, the best it could do would be simply to switch on the radio in the RX mode and wait for the message to arrive. Given that the energy consumption of the OpenMote-B associated with the RX activity is slightly less than the one in TX mode, assuming the same time duration *T*, the node would consume a little bit less.

However, there are several remarks. First of all, considering the omni-directional, global eavesdropper assumed in our work, without decoy messages, a node that does not originate information would wait for the information to arrive for an infinite amount of time, given that the adversary would always disrupt the single packet originated by the source node. Thus, the broadcast delay would be infinite, and so, the energy consumption associated with the completion of the protocol would be lower when adopting SoCİndeed, decoy messages are crucial to create confusion on the adversary side on which transmission to jam, hence enabling the broadcast of the information.

In addition, we remark that, in general, it is not always true that the consumption of the radio in TX mode is higher than the consumption in RX mode. For instance, the CC2420 RF Transceiver (http://www.ti.com/lit/ds/symlink/cc2420.pdf) consumes 17.4 in TX mode and 18.8 mA in RX mode, thus having more energy-savings in the TX mode than in the RX mode. Thus, for the case of the CC2420, even considering the same time duration T, the transmission of decoy messages would not increase the energy consumption.

## 7. Comparison and Final Remarks

In this section, we provide a comparison between SoC and the related work discussed in Section 2. Important remarks are finally included in Section 7.

### 7.1. Comparison

Table 5 summarizes the main features of the protocols discussed in Section 2, as well as the relationship with the proposed SoC scheme with reference to the main requirements assumed in this contribution. The majority of the solutions assume an adversary that is able to listen and jam only a small portion of the network, either considering the frequency spectrum or the geographical region affected by the jammer. Moreover, we observe that none of the cited solutions provide a standard-compatible approach, requiring no modifications at the physical and the MAC layer protocols, and being able to overcome a geographically unlimited wideband reactive jammer. In this context, our contribution is based on a distributed architecture, thus being able to be triggered autonomously, and it can be directly integrated on top of modern IoT communication standards, while being able to guarantee the delivery of the information in the presence of the considered powerful reactive jammer. Finally, it is worth noting that none of the cited contributions experimentally evaluated the energy efficiency of the proposed techniques.

### 7.2. Mitigating Reactive Jamming

Dealing with a reactive jammer is a challenging problem due to the intrinsic assumption of a jammer reacting to the presence of a signal and, therefore, disrupting the message with deterministic effectiveness. Indeed, since it is always reasonable to assume that the jammer has a frequency budget, it is easy for it to compute how many frequencies can be disrupted (in parallel). This means that a reactive jammer is always the winner (preventing all the communications in the network) when the number of concurrently used frequencies is less than the jammer’s frequency budget (*A*). Unfortunately, in a standard setting, this represents the majority of the cases, since the nodes, although they might be assumed as not strictly energy constrained, communicate only when a new event happens, and therefore, the jammer’s budget can be pre-calibrated as a function of the frequency of the specific physical phenomena under the monitoring of the WSN.

With reference to the adversary model assumed in this work and described in Section 3.2, other solutions available in the literature—summarized in Section 2 and Section 7.1—would not be effective. In fact, leveraging the hypothesis that the adversary could jam only some of the available channels, but not all of them, those solutions are based on the transmission of a single message by a single node in the network during a specific time-slot. Given that the adversary assumed in our work is a global eavesdropper, it would be always able to intercept such a transmission and thus to successfully jam it, leading to an infinite broadcast delay.

Our solution, instead, aims at reducing the deterministic effectiveness of the reactive jammer to a probabilistic effectiveness. Indeed, if the number of concurrent transmissions overwhelms *A*, the adversary has to guess which ones to jam. Moreover, assuming all the communications as encrypted and part of them as decoy messages, the adversary cannot determine in advance if its jamming activity will disrupt either a real or a fake message.

## 8. Conclusions

This paper presents SoC, a distributed protocol leveraging decoy messages to guarantee the spreading of information in an IoT network under the presence of a spatially unlimited, global eavesdropping jammer, capable of acting in either a reactive or proactive fashion, and that can disrupt a large fraction of the radio spectrum.

Extensive simulations and real experimental tests show that SoC is effective against the considered adversary model, and if properly configured, it allows the message to be broadcast from a source node to a network constituted by 512 nodes arranged in a random network topology in less than 2000 slots (20 s), even in the presence of a jammer disrupting 80% of the available channels. In addition, SoC has a small footprint (less than 1 kB of code), and it is able to guarantee the broadcast of the information by consuming only 0.18% of the battery capacity, even in the presence of a very powerful adversary, reactively jamming four out of five available channels.

Future work will consider the inclusion of smart cognitive techniques for the assignment of the channels, to minimize the broadcast delay, and the characterization of SoC through a thorough mathematical model.

## Figures and Tables

**Table 1:** Broadcast delay variations compared to the benign scenario (p=0.5).

*A*	*A*/*F*	$B_{d}/B_{d}\left(A=0\right)$
8	0.25	1.32
16	0.5	2
24	0.75	4.09

**Table 2:** ROM and RAM footprint of the implementation of SoC in the OpenWSN protocol stack.

	ROM Footprint (B)	RAM Footprint (B)
SoC	968	8

**Table 3:** Experimental setting: broadcast delay variations compared to the benign scenario.

*A*	*A*/*F*	$B_{d}/B_{d}\left(A=0\right)$
1	0.2	2
4	0.8	6.87

**Table 4:** Components consumption of the OpenMote-B during either TX or RX mode.

Component	Consumption
CPU	13 mA
USB UART	3 mA
LED	1 mA
RX mode	20 mA
TX mode (7 dBm)	34 mA

**Table 5:** Summary of the related work and overview of features of the proposed SoC scheme.

Contribution	Spatially Unlimited Adversary	Reactive Jammer Robustness	No Physical Layer Modifications	MAC-Layer Standard Compliance	Distributed Architecture	Experimental Energy Evaluation
[8]	✓	✕	✓	✓	✕	✕
[9]	✕	✕	✓	✓	✓	✕
[10,11]	✕	✓	✕	✓	✓	✕
[12]	✕	✕	✓	✓	✓	✕
[14]	✕	✓	✕	✕	✓	✕
[15,16]	✕	✓	✓	✓	✕	✕
[18]	✕	✓	✓	✓	✕	✕
[19]	✕	✓	✓	✓	✓	✕
[20]	✓	✓	✕	✕	✓	✕
**SoC**	✔	✔	✔	✔	✔	✔
